# 

**Published:** 1898-01-01

**Authors:** 


					the HOSPITAL, ABSOLUTELY PURE, therefore BEST. Jan. 1st, 1898.
CADBURYS ^ ww CADBURY'S
GOCOA iPl INI COOOA
''SaiSd"i??S5eS,P?'"'"'Pre*'" m> M"m ?ttm> MO ALKALIES USED.
? ?=^^?7Ci.Asr:n>r IVfsren/v lnnhMAHr
No. 588. Vol. XXIII. [SSJIS] Saturday,
Jan. 1st, 1898.
^NURFOLKand NORWICH HOSPI TAL
pnbBcnptio^nas.-j pB<ICE TWOPENCE.

				

